# P-142. Clinical and epidemiological profile of patients with malaria hospitalized at a tertiary level hospital of Central America

**DOI:** 10.1093/ofid/ofae631.347

**Published:** 2025-01-29

**Authors:** Cristel M Rodriguez, Rosa Cárdenas, Felicia Tulloch, Monica Pachar Flores, Jose Suarez, Ana Belen Arauz Rodriguez

**Affiliations:** Hospital Santo Tomás, Panama, Panama, Panama; Hospital Santo Tomas, Panama, Panama, Panama; Hospital Santo Tomás, Panama, Panama, Panama; Hospital Santo Tomás, Panama, Panama, Panama; CIDES Panama; Hospital Santo Tomas, Panama, Panama, Panama

## Abstract

**Background:**

During the last two years Panama has found itself in the midst of a malaria epidemic, with cases spiking by 65% from 4181 in 2021 to 7083 in 2022. As of October 2023, reported cases reached 8301. This situation is thought to be related with human movements of indigenous groups, and the unprecedented number of migrating people crossing Central America through the Darién Gap, a dense rainforest between Colombia and Panama. The increasing incidence of malaria is reflected in hospital admissions in a tertiary level hospital of the city, a non-endemic region of malaria in the country.

**Methods:**

Retrospective study of malaria diagnosed at Hospital Santo Tomás, Panama City, Panama, between 2017 and 2024. Charts from patients hospitalized and confirmed to have malaria were collected. Demographic, clinical and biochemical variables were analyzed using Excell v.2403.

**Results:**

A total of 46 cases were found from 2017 to 2024. The year with more admissions was 2023. Mean age was 34.52 years. Men and women were affected equally. Migrants represented 30% of the cohort and the most common origin was Venezuela. Admissions were more frequent in the months of February and March which coincides with the dry season of the country. The most frequent region to acquire malaria was Darién, in people crossing the Darien Gap or natives from the region. Mean time to onset of symptoms was 11 days and the most common clinical features were fever in 91%, malaise (80%) , and headache (60%). There were 9 cases of severe malaria and one case of cerebral malaria. Only one patient died. In the biochemical profile, the most common findings were thrombocytopenia in 87% and anemia in 76.5%. The diagnosis was made mostly by rapid diagnostic test of malaria (RDT) and thin smear in 52%. The most common *Plasmodium* species was *vivax*, with 43 cases, 2 cases of *falciparum* and 1 case of *falciparum* and *vivax* coinfection. The treatment used for *vivax* malaria was chloroquine and primaquine, and artemether lumefantrine for falciparum malaria. The most common coinfection was dengue fever.

**Conclusion:**

Malaria admissions have been raising in Panama City. The latter raises concern and calls to action to strengthen community networks to diagnose and treat malaria with more laboratory professionals and vector technicians, as well as diagnostic supplies and treatment.

**Disclosures:**

**All Authors**: No reported disclosures

